# A cross-sectional analysis of clinical presentations of and risk factors for enteric protozoan Infections in an Active Duty Population during Operation Iraqi Freedom

**DOI:** 10.1186/s40794-015-0005-6

**Published:** 2015-07-31

**Authors:** John W. Downs, Shannon D. Putnam, David M. Rockabrand, Gamal El Okla, Manal Mostafa, Marshal R. Monteville, Louis E. Antosek, James Herbst, David R. Tribble, Mark S. Riddle, John W. Sanders

**Affiliations:** 1 Uniformed Services University of the Health Sciences, Bethesda, MD USA; 2 Naval Health Research Center, San Diego, CA USA; 3 US Naval Medical Research Unit No. 3, Cairo, Egypt; 4 Marine Wing Support Squadron 273, Al Asad Airbase, Iraq; 5 Forward Deployable Preventive Medicine Unit East, Al Asad Airbase, Iraq; 6 Naval Medical Research Center, Silver Spring, MD USA

## Abstract

**Background:**

Infectious travelers’ diarrhea (TD) is a well-appreciated problem among service members serving abroad, particularly where infrastructure is limited due to ongoing combat operations, and efforts at sanitation and hygiene may not be considered an immediate priority. Bacterial and viral causes of travelers’ diarrhea are well-described among deployed service members, however, gastrointestinal protozoan infections among deployed service members are less well documented. This study’s purpose was to identify potential risk factors for, and clinical presentations of, enteric protozoan infections in an active duty military population deployed to combat operations in the Southwest Asia.

**Methods:**

A cross-sectional study of enteric protozoan infections among US service members deployed in Al-Asad Air Base, Iraq in support of Operation Iraqi Freedom (OIF) was conducted in summer 2004. Subjects were obtained through a randomized sector sampling scheme, and through presentations for care at the air base medical facilities. All study participants provided a stool sample, either diarrhea or solid, upon study entry and completed a questionnaire documenting demographic information, clinical symptoms of any prior diarrheal episodes, and health risk behaviors. Basic diagnostic microscopy for protozoa was conducted to include acid-fast and modified trichrome staining.

**Results:**

Four hundred thirty-seven subjects were included in the analysis, and 75 (17.1 %) subjects were found to have enteric protozoan infections as identified by diagnostic stool microscopy. *Blastocystis hominis* (n = 36), *Entamoeba coli* (n = 25), *Endolimax nana* (n = 20), and *Entamoeba histolytica* (n = 5) were the predominant organisms isolated. Crude incidence of prior episodes of diarrhea was greater among subjects from whom enteric protozoa were isolated compared to those without (IRR 1.66, 95 % CI 1.47-1.87). Bivariate analysis of health risk and hygiene behaviors found increased odds for presence of *Blastocystis hominis* among those service members who reported off base ice (OR 3.61, 95 % CI 1.40-9.28) and raw vegetable consumption (OR 8.18, 95 % CI 1.40-47.5).

**Conclusions:**

This study suggests that US service members deployed to the early stages of OIF were at greater risk of acquiring enteric protozoa than previously understood. The noted prevalence of enteric protozoa among US service members in this study is higher than in prior reports, approaching prevalence expected in the general host nation population, suggesting that US service members operating at Al-Asad Air Base in early OIF were exposed to greater degrees of fecally contaminated food and water, and poor hygienic and sanitation practices. Consumption of food and water prepared by host nation parties in Southwest Asia may place US service members at risk for acquiring intestinal protozoa.

## Background

Infectious diarrhea has remained a problem for service members serving abroad for centuries, particularly in those settings where infrastructure is still limited due to ongoing combat operations, and efforts at sanitation and hygiene may not be considered an immediate priority [1]. Outbreaks of gastrointestinal illness were noted to negatively impact coalition troops during the initial stages of both Operations Iraqi Freedom (OIF) and Enduring Freedom (OEF) [1, 2]. Seventy-six percent of soldiers returning from Iraq or Afghanistan in 2003-2004 reported diarrhea during the deployment with 46 % reporting a decrease in job performance for an average of 2 days [3]. As would be expected, reports of stool samples from service members in the early stages of the most recent war in Iraq yielded pathogens typically associated with infectious diarrhea such as *Shigella*, enterotoxigenic *E. coli* (ETEC), enteroaggregative *E. coli* (EAEC), and norovirus. However, descriptions of gastrointestinal protozoan infections among deployed service members are less well documented. This study’s purpose was to identify potential risk factors for and clinical presentations of enteric protozoan infections in an active duty military population deployed to combat operations in the Southwest Asia, specifically Iraq. This study had two objectives: (1) to characterize the burden of intestinal protozoan infection within an active duty military population performing combat operations in Southwest Asia, and (2) to examine the potential risk factors and health risk behaviors associated with the observed prevalence of intestinal protozoa within this population. An improved understanding of protozoan associations with infectious diarrhea may lead to understanding the risk, optimization of empiric therapy, and identification of potential methods for prevention.

## Methods

### Study site

This study was conducted at Al-Asad Airbase, Iraq in summer 2004. Al-Asad Airbase is an Iraqi Air Force base located in Al Anbar province of western Iraq. At the time of this study, the base was home to approximately 8,000 U.S. service members, predominantly U.S. Marines from marine expeditionary and aviation units. A small hospital and several smaller clinics were established within fixed facilities on the base. Primary food service consisted of two large cafeterias which provided three hot meals per day. The cafeterias were staffed with a significant number of foreign contractors, which is a common practice among military contractors. These facilities were regularly inspected by Navy Preventive Medicine Unit personnel. Additional host nation food vendors were available on base at times, but these sources were not inspected by preventive medicine personnel. Military personnel may have been exposed to off base sources of food and drink during patrols or engagements among the Iraqi populace. Bottled water was available for consumption. Toileting was limited to chemical toilets.

### Study population

Individuals were considered eligible for the study if they were active duty military or a military beneficiary, 18 years or older, had received no antibiotic therapy within the 72 h prior to enrollment (excluding malaria prophylaxis), and had been in Iraq for more than 48 h. Only those subjects for whom stool microscopy results and survey data were available were included in the final analysis.

### Enrollment and follow-up

This study was conducted over a three week period in summer 2004 as part of larger cross-sectional, case finding study that sought to identify cases of infectious diarrhea and asymptomatic controls. A total of 552 subjects were recruited to the study including acute diarrheal cases presenting for medical care, acute diarrheal cases not presenting for medical care (identified during a randomized sector convenience sampling scheme encompassing non-restricted areas of the airbase), and asymptomatic participants (similarly located through the same randomized sector sampling scheme). The random sector sampling scheme was created by dividing the base into 12 sectors, sans restricted areas. Investigators initially randomly selected four sectors a day for three days (via the use of a multi-sided die) for selection of “cases not seeking care” and asymptomatic patients. One of four study teams were sent to each selected sector each day to recruit and enroll subjects through a convenience sampling technique. The rotation of sectors for sampling was continued throughout the study period based on the original three days randomization. Subjects included in the final analysis for this study were those subjects identified in the larger study for which results of stool diagnostic studies for protozoa were available. In total, 437 subjects were included for analysis, of which 111 were symptomatic diarrheal cases at the time of enrollment.

### Specimen collection, processing and testing

All study participants provided a blood sample and a stool sample, either diarrhea or solid, upon study entry and completed a questionnaire documenting demographic information, clinical symptoms of any prior diarrheal episodes, and health risk behaviors. Study questionnaires were collected at the time of stool sample submission. Stool specimens and study questionnaires were placed into a cooler and transported to the laboratory twice each day. All stool samples underwent microbiologic analysis for bacterial, viral, and protozoan causes as part of the larger cross-sectional study. Basic diagnostic microscopy for protozoa was conducted by NAMRU-3 staff to include acid-fast and modified trichrome staining.

### Study definitions

A case of enteric protozoa infection was defined by the direct visualization on diagnostic stool microscopy of protozoal cysts or trophozoites from the stool specimen submitted. This case definition was utilized both for those subjects with acute diarrhea at the time of enrollment, and for those subjects that were asymptomatic on enrollment.

### Data management

Study stool samples and questionnaires were linked by a unique identification number (UID) assigned to each subject. This was done to ensure and maintain the confidentiality of study subjects. The UID was included on all questionnaires, clinical forms, stool samples, and laboratory forms. All data was entered and verified using Microsoft Access database.

### Statistical analysis

Subjects from whom enteric protozoa were identified were compared to those with no enteric protozoa identified from stool samples. Due to the frequency in which it was identified, additional analysis was conducted comparing those subjects from whom only *Blastocystis hominis* was isolated to those subjects with no enteric protozoa isolated from stool. Univariate analysis was conducted using standard statistical tests. Comparisons of median age, median time in theater, and median time at Al-Asad were conducted with Wilcoxon Rank Sum tests. Testing of independent categorical variables was conducted with either Pearson’s chi-square test or Fisher’s Exact test. Crude incidence rates of prior episodes of diarrhea were calculated using the number of self-reported prior episodes of diarrhea during the deployment, and the self-reported days in theater. Poisson regression was used to estimate incidence rates and confidence intervals, and crude incidence rate ratios. Health risk behavior survey responses in some cases utilized Likert scales. For analysis, these responses were dichotomized so that responses of “always” or “frequently” were treated as “yes”, and those responses indicating “rarely” or “never” were treated as “no.” Likert responses of “sometimes” were removed from analysis. Unadjusted odds ratios were obtained through bivariate logistic regression analysis to assess the strength of association between individual health risk behaviors and the presence of enteric protozoa. All data analysis was conducted using Stata/IC Version 12.1, (StataCorp, College Station, TX) and statistical significance was set at *p* < 0.05.

### Ethical compliance

This study (DOD# NAMRU3.2004.0011) was reviewed and approved by the Institutional Review Board of the US Naval Medical Research Unit No. 3 in compliance with all Department of Navy Federal regulations governing the protection of human subjects. Informed consent was obtained and documented from all adult participants.

## Results

This study determined there were a total of 437 observations available for analysis. There were no significant differences found by median age, median days in theater, or median days at Al-Asad Airbase when comparing subjects with enteric protozoa to those without protozoa isolated (Table 1). No differences in distribution by military rank, race, or branch of service were found. Overall, the subjects were found to have a median age of 23 years, and had approximately 4 months in theater (median = 126 days) as well as at Al-Asad (median = 112 days). As expected, the overwhelming majority of subjects were enlisted (96.5 %), male (97.7 %), and members of the United States Marines Corps (86 %).

**Table 1:** Demographics of Study Population

		Protozoa Found	No Protozoa Found	Total	p value
		n = 75	n = 362	n = 437	
Male, n (%)		74 (98.6)	353 (98.0)	427	0.72
Median Days at Al Asad (IQR)		112 (93-118)	112 (101-120)	113 (101-121)	0.44
Median Days in Theater (IQR)		126 (115-130)	126 (117-130)	127 (120-132)	0.89
Median Age (IQR)		23.0 (21-27)	23.0 (21-27)	23.0 (21-27)	0.95
Age, n (%)					
	<20	4 (5.3)	30 (8.3)	34 (7.8)	
	20-29	57 (76)	258 (71.2)	315 (72.0)	
	30-39	10 (13.3)	56 (15.4)	66 (15.1)	
	40-49	4 (5.3)	16 (4.4)	20 (4.5)	
	>50	0 (0)	2 (0.5)	2 (0.5)	0.81
Rank, n (%)					
	E1-E3	36 (48.0)	159 (44.1)	195 (44.8)	
	E4-E9	36 (48.0)	189 (52.5)	225 (51.7)	
	Officers	2 (2.7)	11 (3.0)	13 (3.0)	
	Civilian	1 (1.3)	1 (0.2)	2 (0.5)	0.58
Race, n (%)					
	African American	3 (4.1)	29 (8.2)	32 (7.5)	
	Caucasian	52 (71.2)	212 (60.5)	264 (62.4)	
	Hispanic	15 (20.5)	76 (21.7)	91 (21.5)	
	Other	3 (4.1)	33 (9.4)	36 (8.5)	0.21
Service, n (%)	Marines	67 (89.3)	309 (85.3)	376 (86.0)	
	Navy	8 (10.7)	48 (13.2)	56 (12.8)	
	Army	0 (0)	5 (1.4)	5 (1.1)	
Active Component, n (%)		67 (89.3)	332 (91.7)	399 (91.3)	0.23
Malaria Prophylaxis, n (%)		66 (88.0)	305 (84.9)	317 (85.5)	0.86

Enteric protozoa were isolated from 75 (17.1 %) stool samples. *Blastocystis hominis* (n = 36), *Entamoeba coli* (n = 25), and *Endolimax nana* (n = 20), made up 90 % of all identified protozoa with multi- protozoan co-infections included (Table 2). Fourteen subjects were found to have multi- protozoan co-infections (Table 3). No helminths were identified from any stool samples. It is important to note here that the majority of identified protozoa were non-pathogenic organisms, although the pathogenicity of *Blastocystis hominis* remains controversial even at present. Only six subjects were identified to have true infections with enteric protozoa widely considered to be pathogenic, *Entamoeba histolytica* (n = 5) and *Giardia lamblia* (n = 1).

**Table 2:** Distribution of Enteric Protozoa

Protozoa Distribution	n (%)
*Blastocystis hominis*	27 (36)
*Entamoeba coli*	19 (25.3)
*Endolimax nana*	12 (16)
*Entamoeba histolytica*	1 (1.3)
*Iodamoeba butschlii*	1 (1.3)
*Giardia lamblia*	1 (1.3)
Multi protozoa infections	14 (18.7)
Total	75

**Table 3:** - Multi-protozoa Coinfections

			n
*Blastocystis hominis*	*Chilomastix mesnili*		1
*Blastocystis hominis*	*Endolimax nana*		3
*Blastocystis hominis*	*Entamoeba coli*		4
*Blastocystis hominis*	*Entamoeba histolytica*	*Endolimax nana*	1
*Endolimax nana*	*Chilomastix mesnili*		1
*Endolimax nana*	*Entamoeba coli*		1
*Endolimax nana*	*Entamoeba histolytica*		2
*Entamoeba histolytica*	*Entamoeba coli*		1
Total			14

Analysis of associated symptoms and health risk behaviors at the time of study enrollment found a trend toward greater reported prevalence of current diarrhea among those without protozoa identified (27.1 %) compared to those subjects with protozoa identified (17.3 %; *p* = 0.08). There were no differences noted in prevalence of reported current fever. Among those who reported diarrhea previously during the deployment, there were no significant differences in median number of prior episodes (3), and no significant differences in diarrhea associated symptoms. A near significant (*p* = 0.07) greater proportion of subjects with protozoa identified reported having previous episodes of diarrhea of such severity that back-up personnel were required to come in to cover the subject’s work shift. Additionally, near significant differences were noted in reporting off base travel in the prior 5 days. Subjects without protozoa identified tended to more frequently report off base travel in the last 5 days (*p* = 0.06) (Table 4).

**Table 4:** Characteristics of prior diarrhea during deployment

	Protozoa Found	No Protozoa Found	Total	p value
Had diarrhea at study enrollment, n (%)	13 (17.3)	98 (27.1)	111 (25.4)	0.08
Fever present at study enrollment, n (%)	1 (1.3)	4 (1.1)	5 (1.1)	0.87
Had other diarrhea episodes during the same deployment	52 (69.3)	230 (63.5)	282 (64.5)	0.34
For Any previous diarrheal episode:				
Median # of loose stools/day for any episode (IQR)	4 (3-6)	5 (3-7)	5 (4-7)	0.48
Abdominal Cramps	34 (65.4)	169 (73.4)	203 (72.0)	0.24
Nausea	24 (46.1)	101 (44.3)	125 (44.6)	0.81
Vomiting	6 (11.8)	38 (16.7)	44 (15.7)	0.38
Headache	24 (46.1)	102 (44.5)	126 (44.8)	0.83
Muscle Aches	18 (34.6)	72 (31.5)	90 (32.1)	0.67
Fevers	11 (21.1)	32 (14.1)	43 (15.4)	0.20
Blood in Stool	5 (9.6)	17 (7.4)	22 (7.8)	0.60
Visited Sick Call	16 (30.1)	46 (20.3)	62 (22.2)	0.12
Hospitalized?	1 (1.9)	4 (1.8)	5 (1.8)	0.96
Back Up Called	5 (9.6)	8 (3.5)	13 (4.6)	0.07

Bivariate analysis suggested that those who reported having drinks with ice or eating raw vegetables with meals while off base were at increased odds of having protozoa identified in their stool samples, although results were not significant (Table 5). Point estimates suggest that protozoa-positive subjects were at over 3.5 times greater odds of reporting that they ate raw vegetables off base (OR 3.70 95 % CI 0.79-3.71). No other significant associations were noted between reported health risk behaviors and subjects with protozoa identified in stool. When crude incidence rates were calculated, subjects with enteric protozoa reported 3.97 (95 % CI 3.31-4.78) prior episodes of diarrhea per 100 person-days in theater, compared to a rate of 2.39 (95 % CI 2.25-2.55) per 100 person-days among protozoa-negative subjects. This resulted in a crude incidence rate ratio of 1.66 (95 % CI 1.47-1.87), indicating that protozoa-positive subjects developed prior episodes of diarrhea at a rate 66 % higher than protozoa-negative subjects in the days after arrival to theater, but prior to study enrollment.

**Table 5:** Bivariate Analysis for Risk Factors for Stool Protozoa Detection

	Odds Ratio	95 % CI
Age		
<20	1.00	
20-29	1.65	0.56-4.88
30-39	1.33	0.38-4.63
40-49	1.87	0.41-8.51
Rank		
E1-E3	1.00	
E4-E9	0.84	0.50-1.39
Officers	0.80	0.17-3.78
Civilian	4.41	0.26-72.2
Race		
African American	1.00	
Caucasian	2.37	0.69-8.08
Hispanic	1.90	0.51-7.08
Other	0.87	0.16-4.69
Service		
Marines	1.00	
Navy	0.76	0.34-1.69
Current Diarrhea		
No	1.00	
Yes	0.56	0.29-1.07
Diarrhea on prior deployment		
No	1.00	
Yes	0.83	0.49-1.40
Diarrhea previously on this deployment		
No	1.00	
Yes	1.30	0.76-2.21
Off Base Travel Last 5 Days		
No	1.00	
Yes	0.48	0.22-1.03
Ate Off Base Last 5 Days		
No	1.00	
Yes	0.68	0.27-1.71
Ate On Base Local Last 5 Days		
No	1.00	
Yes	1.07	0.57-1.98
When eating off base, I have drinks with ice.		
No	1.00	
Yes	1.71	0.79-3.71
When eating off base, I eat raw (uncooked) vegetables		
No	1.00	
Yes	3.70	0.80-16.90
I eat off base		
No	1.00	
Yes	1.04	0.34-3.19
I wash my hands before eating		
No	1.00	
Yes	1.01	0.11-8.81
I wash my hands after using the latrine		
No	1.00	
Yes	1.35	0.29-6.15

Of the 75 subjects from which protozoa were identified, *Blastocystis hominis* was the only protozoa identified in the stool samples of 27 subjects (Table 2). Twenty-one of these 27 subjects (77.8 %) denied having diarrhea at the time of study enrollment, and only 1 (3.7 %) reported current fever at the time of study enrollment (Table 6). Fifteen of the 27 subjects with *Blastocystis hominis* only identified reported diarrhea during the present deployment, and answered survey questions related to these prior episodes. Among the 15 subjects with only *Blastocystis hominis* identified who reported diarrhea during the present deployment, associated symptoms included nausea in ten (67 %), abdominal cramps in nine (60 %), headache in nine (60 %), fever in five (33 %), blood in stool in two (13.3 %), and vomiting in one (6.7 %). These proportions are not significantly different from those seen in subjects without protozoa identified. However, a greater proportion of subjects with *Blastocystis hominis* reported needing to have a backup called to cover their shift due to gastrointestinal upset compared to those subjects without protozoa identified (20 % vs 3.5 %; p = 0.02).

**Table 6:** Characteristics of Blastocystis sp. Diarrhea

	*Blastocystis* Only Isolated n (27)	No Protozoa Isolated n (362)	p value
Diarrhea at study enrollment, n(%)			
Yes	6 (22.2)	98 (27.1)	
No	21 (77.8)	263 (72.9)	0.58
Fever at study enrollment, n(%)			
Yes	1 (3.7)	4 (1.1)	
No	26 (96.3)	356 (98.9)	0.25
Diarrhea on Prior Deployments			
Yes	4 (14.8)	131 (36.3)	
No	23 (85.1)	230 (63.7)	0.03
Had other diarrhea episodes during this deployment			
Yes	15 (55.6)	230 (63.5)	
No	12 (44.4)	132 (36.4)	0.41
Date of last diarrhea			
Less than 1 week ago	4 (25)	60 (26.4)	
1-2 weeks ago	7 (43.8)	65 (28.6)	
3-4 weeks ago	3 (18.8)	46 (20.2)	
>4 weeks ago	1 (6.2)	56 (24.7)	0.05
Characteristics of prior diarrhea episodes during this deployment			
Abdominal Cramps			
Yes	9 (60)	169 (73.5)	
No	6 (40)	61 (26.5)	0.25
Nausea			
Yes	10 (66.7)	101 (44.3)	
No	5 (33.3)	127 (55.7)	0.09
Vomiting			
Yes	1 (6.7)	38 (16.7)	
No	14 (93.3)	190 (83.3)	0.30
Headache			
Yes	9 (60)	102 (44.5)	
No	6 (40)	127 (55.50)	0.24
Muscle Aches			
Yes	6 (40)	72 (31.6)	
No	9 (60)	156 (68.4)	0.50
Joint Pain			
Yes	3 (20)	44 (19.4)	
No	12 (80)	183 (80.6)	0.95
Fevers	5 (33.3)	32 (14.1)	
Yes	10 (66.7)	195 (85.9)	0.06
No			
Blood in Stool			
Yes	2 (13.3)	17 (7.5)	
No	13 (86.7)	211 (92.5)	0.41
Visited Sick Call			
Yes	3 (18.8)	46 (20.4)	
No	13 (81.2)	180 (79.7)	0.88
Admitted?			
Yes	1 (6.2)	4 (1.8)	
No	15 (93.8)	221 (98.2)	0.23
Back Up Called			
Yes	3 (20)	8 (3.5)	
No	12 (80)	221 (96.5)	0.02
Off Base Travel Last 5 Days			
Yes	1 (6.7)	70 (30.4)	
No	14 (93.3)	160 (69.6)	0.07
Ate Off Base Last 5 Days			
Yes	2 (13.3)	37 (16.1)	
No	13 (86.7)	193 (83.9)	0.78
Ate On Base Local Last 5 Days			
Yes	6 (40)	85 (37.0)	
No	9 (60)	145 (63.0)	0.81

Bivariate analysis (Table 7) comparing subjects with only *Blastocystis hominis* identified to those subjects without protozoa identified found that subjects with *Blastocystis hominis* were at about 70 % lesser odds of reporting diarrhea on a prior deployment (OR 0.30, 95 % CI 0.10-0.90). Twenty-three (85 %) of 27 subjects with *Blastocystis hominis* only identified denied having had diarrhea on prior deployments. Point estimates also suggested a trend toward lesser odds of having traveled off base in the last 5 days for those subjects with *Blastocystis hominis* only identified (OR 0.16, 95 % CI 0.02-1.27). Most notably, subjects with *Blastocystis hominis* only identified reported over three times greater odds of using ice in drinks off base (OR 3.61, 95 % CI 1.40-9.28), and over eight times greater odds of having eaten raw vegetables off base (OR 8.18, 95 % CI 1.40-47.5).

**Table 7:** Bivariate Analysis for Risk Factors for Blastocystis sp. Detection

	Odds Ratio	95 % CI
Age		
<20	1.00	
20-29	1.10	0.24-4.97
30-39	0.80	0.12-5.00
40-49	2.80	0.42-18.60
Rank		
E1-E3	1.00	
E4-E9	0.98	0.44-2.18
Officers	1.20	0.14-10.10
Service	1.00	
Marines	0.80	0.23-2.77
Navy		
Current Diarrhea		
No	1.00	
Yes	0.76	0.30-1.95
Diarrhea on prior deployment		
No	1.00	
Yes	0.30	0.10-0.90
Diarrhea previously on this deployment		
No	1.00	
Yes	0.71	0.32-1.57
Date of last diarrhea		
Less than 1 week ago	1.00	
1-2 weeks ago	1.61	0.45-5.79
3-4 weeks ago	0.97	0.20-4.59
>4 weeks ago	0.27	0.03-2.47
Off Base Travel Last 5 Days		
No	1.00	
Yes	0.16	0.02-1.27
Ate Off Base Last 5 Days		
No	1.00	
Yes	0.8	0.17-3.70
Ate On Base Local Last 5 Days		
No	1.00	
Yes	1.13	0.39-3.30
I have drinks with ice off base.		
No	1.00	
Yes	3.61	1.40-9.28
Eat raw (uncooked) vegetables off base vegetables		
No	1.00	
Yes	8.18	1.40-47.5
I eat off base		
No	1.00	
Yes	1.56	0.34-7.20

## Discussion

Multiple prior reports have addressed the impact of infectious diarrhea on combat operations during Operation Iraqi Freedom (OIF). However, limited references have been made to identified enteric protozoa during either OIF or the prior war in Iraq. Thornton *et al.*’s report on gastroenteritis in U.S. Marines during the initial invasion of Iraq mentions only a single case of diarrhea of protozoan etiology (*Cryptosporidium*) in 129 stool samples obtained within southern Iraq [2]. However, Thornton’s study was conducted in the weeks just after the initial invasion period in spring 2003. Thus, subjects had been in Iraq for a shorter duration than in our study, and had been consuming primarily U.S. rations. In contrast, Monteville *et al.*’s analysis of service members arriving in Qatar for “rest and relaxation” leave from Iraq or Afghanistan in 2006 found enteric protozoa in as many as 20 % of those service members with diarrhea [4]. Enteric protozoa were identified in about 16 % of subjects arriving from OIF, most commonly *Blastocystis hominis*, *Entamoeba coli*, and *Endolimax nana*. The Monteville *et al.* study did include members with longer deployments which may have contributed to the greater prevalence of intestinal protozoa. This suggested perhaps a greater role of gastrointestinal protozoa in diarrhea among deployed service members than previously considered, particularly on extended deployments. Historically, Hyams and colleagues collected over 400 stool samples from service members with gastroenteritis during deployment to Saudi Arabia in preparation for the First Gulf War with Iraq. Their analysis of these samples found a bacterial pathogen in about half of all submitted samples, but found no evidence of intestinal protozoa in any of the over 400 samples [5]. Malone reported in 1991 on a separate sample of over 400 stool samples obtained from U.S. Marines returning for Operation Desert Shield/Desert Storm, and found *Giardia* cysts in only 9 specimens with no evidence of other intestinal protozoa [6]. Of note, subjects in Malone’s study had apparently entered Iraq as part of combat operations during Operation Desert Storm, but subjects in Hyams’ study only served in Saudi Arabia for Operation Desert Shield. All of the above studies appear to have used similar diagnostic microscopy techniques for identification of enteric parasitic (protozoa and helminths).

Looking at deployments to the Middle East outside the Iraq theater, a 1991 report on almost 200 stool samples collected from service members with acute diarrhea during a deployment to Egypt also did not find evidence of intestinal protozoa in these samples [7]. A study conducted in 2002 at a U.S. base in Turkey found intestinal protozoa in about 4 % of cases of acute travelers’ diarrhea [8]. Finding few intestinal protozoa in samples collected for acute diarrheal episodes may be expected as enteric protozoa are less frequent causes of acute diarrhea, but have traditionally been more associated with chronic or persistent diarrhea [9]. A 2011 study did report a 23% prevalence of protozoa among 72 stool samples collected from a peacekeeping force in the Sinai Peninsula of Egypt in 2004-2005 [10]. This 2011 study of forces in Egypt also reported *Blastocystis hominis*, *Entamoeba coli*, and *Endolimax nana* as the most commonly identified enteric protozoa. This study also appears to confirm that the most frequently isolated protozoa among US service members deployed to the Middle East are *Blastocystis hominis*, *Entamoeba coli*, and *Endolimax nana* as similarly noted in two prior studies [4, 10]. It is worthwhile to reinforce here that of almost all identified protozoa in this study are non-pathogenic organisms, such as *Entamoeba coli*, and *Endolimax nana*. The pathogenicity of *Blastocystis hominis* remains controversial up to the present, and would have been considered a commensal at the time of the study [11].

The prevalence of enteric protozoa in this study is high and suggests that sanitary conditions for US service members at the time of this study were less than optimal. The reported compliance with appropriate hygiene measures of washing hands before eating was quite high (98.4 % of all subjects answered ‘yes’), as well as washing hands after toileting (95.9 % of all subjects answered ‘yes’), although, these types of questions may introduce a bias, (*e.g.*, social desirability bias), and compliance with these and other proper hygiene practices may well have been much lower. The consumption of off base ice which presumably would have been provided in an off base restaurant or a setting where prepared food was provided indeed had the greatest associations with the presence of *Blastocystis hominis* in stool.

This significant proportion of *Blastocystis hominis* infection (7.5 % of all subjects) in US service members has not been previously reported. Results in this study would at least suggest a greater than expected prevalence of *Blastocystis hominis* among a group of generally healthy deployed US service members. Those subjects with only *Blastocystis hominis* identified in their stool did not report significant differences in gastrointestinal symptoms compared to those subjects with no enteric protozoa identified. However, the small number of *Blastocystis hominis* cases that reported associated symptoms of prior diarrhea (only 15 of 27 answered survey questions regarding prior episodes of diarrhea) limits the ability to detect any real differences. Significant controversy remains in the current literature as to the true pathogenicity of *Blastocystis hominis*, and the need to treat when the organism is identified in stool [11–13]. While gastrointestinal symptoms for subjects with *Blastocystis hominis* infection did not differ significantly from those subjects without enteric protozoa, the finding that subjects with only *Blastocystis hominis* isolated more frequently reported having had a back-up called to cover their shift suggests that whatever symptoms were present were possibly more severe among those with *Blastocystis hominis*. This may be another indication of the potential pathogenicity of *Blastocystis hominis,* at least among service members who were presumed to have acquired the protozoa upon arrival into theater. More likely, however, is that the presence of *Blastocystis hominis* among those requesting back-up may simply be indicative of a prior ingestion of food or drink with heavy fecal and multi-pathogen contamination, to include viral and bacterial causes of acute diarrhea.

The finding of a trend toward a greater proportion of subjects with protozoa identified reporting no off base travel in the prior five days may be the result of gastrointestinal infection or illness acquired during previous off base travel. Perhaps, these symptoms of these illnesses prevented service members from participating in off base operations in the days immediately prior to study entry. The finding of greater incidence of episodes of diarrhea among those subjects with protozoa identified suggests that those subjects may have had ongoing or recurrent episodes of diarrhea rather than acute episodes which subsequently resolved as may be expected with an acute bacterial diarrheal infection. Again, these recurrent episodes may have contributed to service members having remained on base in the five days prior to study entry. This may be further evidenced by the finding of a trend toward greater odds of having required a back-up to be called in to cover a service member’s shift among those with protozoa identified.

This study has a number of notable limitations. It is a cross-sectional study and therefore subject to the limitations of all cross-sectional studies. Most notably, we are unable to clearly establish a causal association between reported health risk behaviors and presence of enteric protozoa as all information was collected simultaneously. We have limited information regarding where study subjects may have traveled while en route to Iraq, or how long they may have remained at those locations. Some service members did report prior deployments to a variety of locations, and it is likely that some service members may have been on shipboard deployments with en route ports of call prior to their arrival in Iraq. We are unable to determine which, if any, subjects may have arrived to theater already carrying enteric protozoa, though this is unlikely. We are confident in our ability to discern *Entamoeba coli, Endolimax nana*, and *Entamoeba histolytica* by diagnostic microscopy, but recognize that some may note the possibility of misidentifying one species for another. Regardless of this possibility for misidentification, the presence of any of these organisms would indicate fecal contamination of food and/or water sources, and may indicate risk for other pathogenic enteric protozoa. For this study, we were not able to consider the possibility of bacterial or viral co-pathogens that may have contributed to the clinical presentation of study participants. Furthermore, this study is not a comprehensive assessment for enteric protozoa as only direct microscopy was used for identification. More sensitive protozoan identification methods are now available and FDA-approved, such as enzyme immunoassays for *Entamoeba histolytica* and *Giardia*, and polymerase chain reaction for *Cryptosporidium* and *Giardia* [14]. This study population was also almost entirely male enlisted U.S. Marines; thus, any potential gender specific differences in protozoa presence or risk factors for protozoa presence were unable to be explored in this study.

This study also has several strengths. To our knowledge, this is the only study to date specifically exploring the prevalence of enteric protozoa among service members deployed to Operation Enduring Freedom or Operation Iraqi Freedom. The prior reports that have included protozoa etiologies of acute diarrhea among service members deployed to Southwest Asia have not attempted to link diarrheal associated symptoms with enteric protozoa presence. Our study was able to combine information on health risk behaviors, associated clinical symptoms, and stool diagnostic microscopy. Additionally, unlike any prior study among deployed US service members, this study was also able to associate health risk behavior information with stool samples positive for *Blastocystis hominis*. Our results suggest that the presence of *Blastocystis hominis* is associated with greater lost work days, but we are unable to conclude at this time whether these lost work days are attributable to *Blastocystis hominis* directly, or if its presence is simply indicative of exposure to a heavy, and potentially diverse, pathogen load previously.

## Conclusions

This is the only study to date that attempts to determine health risk behavior associations specifically with the presence of enteric protozoa among deployed US service members. This study found strong associations with *Blastocystis hominis* presence in stool samples and prior reported use of ice in off base drinks, and consumption of raw vegetables when eating off base during the deployment. Similar, but not statistically significant, results were noted when all protozoa were pooled for analysis. This study reiterates the need for deployed US service members to consume food and water only from sources that have been approved by US preventive medicine and public health officials. Consumption of food and water prepared by host nation parties will likely place US service members at risk for acquiring intestinal protozoa. The noted prevalence of enteric protozoa among US service members in this study is higher than in prior reports, approaching prevalence expected in the general host nation population, which suggests that US service members operating at Al-Asad Air Base in early stages of Operation Iraqi Freedom were exposed to greater degrees of fecally contaminated food and water, and poor hygienic and sanitation practices. Future studies should consider the impact that intestinal protozoa have on unit readiness, and the clinical effects of *Blastocystis hominis* infections on deployed US service members. Study subjects were not followed beyond the study window; therefore, we are unable to comment on the potential long term gastrointestinal consequences of these protozoan exposures. Future work should also consider the possibility of extended alteration of gut flora following exposure to enteric protozoa.
